# Salvianolic acid B induced upregulation of miR-30a protects cardiac myocytes from ischemia/reperfusion injury

**DOI:** 10.1186/s12906-016-1275-x

**Published:** 2016-09-01

**Authors:** Dan Li, Jun Wang, Jincai Hou, Jianhua Fu, Jianxun Liu, Ruichao Lin

**Affiliations:** 1 Beijing Key Lab of Chinese Materia Medica Quality Evaluation, School of Chinese Pharmacy, Beijing University of Chinese Medicine, 6 Wngjing Zhonghuan Nanlu, Chaoyang District, Beijing, 100102 China; 2 Institute of Basic Theory, China Academy of Chinese Medical Sciences, Beijing, 100700 China; 3 Institute of Basic Medical Sciences of Xiyuan Hospital, Beijing Key Laboratory of Pharmacology of Chinese Materia Medicine, Beijing, 100091 China

**Keywords:** Ischemic heart disease, Salvianolic acid B, Autophagy, miR-30a

## Abstract

**Background:**

MicroRNAs (miRNAs) are a novel class of powerful, endogenous regulators of gene expression. This study was designed to ascertain if miR-30a is involved in the cardioprotective actions of salvianolic acid B (Sal B) against myocardial ischemia–reperfusion (I–R) injury through suppression of autophagy.

**Methods:**

Murine myocardial cells that had undergone primary culture were induced by I–R and incubated with Sal B (25, 50, 100 μM) in the presence of a miR-30a mimic or miR-30a inhibitor. Expression of miR-30a, beclin-1, LC3-II and p-Akt protein, cell viability, and lactic acid dehydrogenase (LDH) release were assessed.

**Results:**

miR-30a expression was down-regulated remarkably in I–R cells, and this suppression could be reversed by Sal B in a dose-dependent manner. Sal B repressed autophagy in I–R myocardial cells. Sal B improved cell viability and reduced the rate of LDH leakage, which suggested that autophagy suppression was beneficial for cell survival. Knockdown of miR-30a with a miR-30a inhibitor could reverse the anti-autophagy effect of Sal B against I–R injury. Furthermore, we confirmed that Sal B has a protective role in miR-30a-mediated autophagy through the PI3K/Akt signaling pathway, which was abrogated by the PI3K inhibitor LY294002.

**Conclusions:**

These data suggest that miR-30a is involved in Sal B-mediated cardioprotection against I–R injury through the PI3K/Akt signaling pathway.

**Electronic supplementary material:**

The online version of this article (doi:10.1186/s12906-016-1275-x) contains supplementary material, which is available to authorized users.

## Background

Despite optimal treatment, ischemic heart disease - is the leading cause of death worldwide [1], and the second leading cause of cardiovascular death in China [2]. Treatment for myocardial ischemia includes cholesterol-lowering medications, beta-blockers, nitroglycerin, and calcium antagonists [3–5]. Such treatment can attenuate myocardial infarction, reduce cardiomyocyte apoptosis and restore contractile dysfunction. Recently, constituents from natural herbs have attracted attention with regard to pharmaceutical development.

MicroRNAs (miRNAs) are endogenous small RNA molecules best known for post-transcriptional gene regulation. MiR-30a is a member of the miR-30 family, which was identified significantly altered miRNAs in mice cardiac tissue with a model of myocardial I/R compared with normal cardiac tissue in our previous study. Other Studies also reported that miR-30a was found to be one of the differentially expressed miRNAs involved in cardiovascular pathophysiology [6–9].

Autophagy is a highly conserved cellular mechanism that plays a key part in the turnover of long-lived proteins, RNA, and dysfunctional organelles [10]. In mouse hearts [11] and isolated rabbit hearts [12], autophagy can be induced by ischemia and enhanced further by reperfusion. Activation of autophagy is reflected by an increase in the abundance of key proteins of autophagy-related pathways: beclin-1, light chain 3 (LC3), autophagy-related gene 5-12 complex (ATG5-12), and p62 [13–15]. Therefore, regulation of autophagy by pharmacologic approaches is a potential strategy to treat heart diseases. In recent years,the close relationship between miR-30a and autophagy has been observed.. Studies have shown that miR-30a can negatively regulate expression of the beclin-1 gene, resulting in decreased autophagic activity in cancer cell lines such as T98G, MDA-MB-468 and H1299 [16]. Circulating miR-30 has been shown to be positively associated with left ventricular wall thickness, and regarded to be an important marker for the diagnosis of left ventricular hypertrophy due to miR-30a-induced alterations in expression of the beclin-1 gene and autophagy in cardiomyocytes [8]. Activation of the phosphoinositide 3-kinase (PI3K)/Akt signaling pathway, which could inhibit autophagy, is related to cardiomyocyte protection [17]. Hu et al. [18]. also reported that inhibition of the PI3K/Akt signaling pathway could abolish the effects of B-type natriuretic peptide on myocardial ischemia–reperfusion (I–R) injury. It has been reported that PI3K catalytic subunit delta is a direct target of miR-30a because miR-30a binds directly to the 3′-UTR of PI3K catalytic subunit delta mRNA [19]. In addition, the PI3K/ Akt) the mammalian target of Rapamycin (mTOR) signalling pathway negatively regulate autophagy under certain conditions [20].

Salvianolic acid B (Sal B) is the most important and abundant bioactive component of the traditional Chinese herb *Salvia miltiorrhiza*. Sal B is used widely to treat cardiovascular diseases [21]. Recent data have shown that Sal B protects the myocardium and cardiovascular system through: inhibition of expression of matrix metalloproteinase-9 and fibrosis [22]; inhibition of adenosine diphsophate-induced platelet aggregation [23]; suppression of expression of intercellular adhesion molecule-1 in tumor necrosis factor-α-treated endothelial cells [24]. Evidence also suggests that Sal B inhibits autophagy and protects starving cardiomyocytes [25].

In summary,both miR-30a and Sal B have a close relationship with cardiovascular disease through autophagy.but few studies have focused on the mechanism of action of miR-30a in the cardioprotective effects of Sal B. We hypothesized that Sal B may have a more important role in cardiovascular disease than currently thought. Hence, to examine the involvement of the miR-30a/PI3K/Akt pathway in ischemic cardiovascular injury and the potential protective effect of Sal B in terms of autophagy, we used an in vitro model of I–R to observe the effect of Sal B.

## Methods

### Reagents and antibodies

Reagents associated with cell culture were obtained from Invitrogen (Carlsbad, CA, USA). Cell Counting Kit 8 (CCK-8, CK04) was purchased from Dojindo Laboratories (Kumamoto, Japan). Lactic acid dehydrogenase (LDH) Cytotoxicity Assay kit was obtained from Gen Way Biotech (San Diego, CA, USA). LY294002 (PI3K inhibitor) was purchased from Sigma–Aldrich (Saint Louis, MO, USA). A double-stranded pre-miR-30a mimic and a single-strand inhibitor were obtained from GenePharma (Shanghai, China). Pre-miR-30a is chemically modified to guide the selection and stability of strands. The negative control for pre-miRs was used as a non-sensical oligonucleotide control. Antibodies against beclin-1, LC3-II, p-Akt (Ser 473) and total-Akt were purchased from Cell Signaling Technology (Danvers, MA, USA). Sal B (purity ≥ 98 %) was obtained from the National Institutes for Food and Drug Control (Beijing, China).

### Cell culture and miR-30a transfection

One-day-old male C57BL/6 J mice (certificate no. SCXK (Jing) 2012-0001) were purchased from Vital River Laboratory Animal Technology (Beijing, China). All experiments with animals were performed in accordance with China Academy of Chinese Medical Sciences Guide for Laboratory Animals that conforms to the Guide for the Care and Use of Laboratory Animals published by the U.S. National Institutesof Health. The protocol was approved by the Institute’s Animal Care and Use Committee of Experimental Animal Research Institute, China Academy of Chinese Medical Sciences where there were veterinarians and scientists involved who are qualified in the evaluation of animal ethical issues.

To acquire primary myocardial cells, separated ventricular tissue was minced into small pieces followed by digestion with 0.05 g/L collagenase II–0.625 g/L trypsin at 37 °C for 20 min. The suspension was filtered through a 200-μm mesh sieve and resuspended in myocardial cell-complete medium (Dulbecco’s modified Eagle’s medium (DMEM) supplemented with 10 % fetal bovine serum (FBS), 1 × 10^5^ U/L penicillin, 1 × 10^5^ U/L streptomycin sulfate, pH 7.2. The unattached cells were plated at 5 × 10^4^ cells/cm^2^ on appropriate culture dishes or plates. The subsequent experiments were performed 48-72 h after plating, when the cardiomyocytes of mice were cultured to 70-80 % confluence.miR-30a inhibitor (100 nmol/L), miR-30a mimics (100 nmol/L) or negative control (100 nmol/L) were transfected into myocardial cells for 24 h using siRNA-MATE (GenePharma) according to manufacturer instructions.

### I–R and treatment with Sal B

An oxygen–glucose deprivation (OGD) model was used to mimic ischemia according to the method established by Wang et al. [21]. Briefly, myocardial culture medium was substituted with glucose-free DMEM. Culture flasks (or plates) were placed in a sealed tank with persistent low flow (1.5 L/min) of a mixture of 95 % N_2_ and 5 % CO_2_. The tank was placed in an incubator at 37 °C for 2 h to mimic ischemia. After ischemia 2 h, mimic reperfusion injury was achieved by changing the medium in normal culture fluid and exposing cells to ambient air, and the cells were maintained for another 2,6,and 24 h.

Drug treatment involved cells being incubated with 25 μM, 50 μM and 100 μM Sal B at the onset of ischemia and reperfusion. Treatment of myocardial cells with 100 μM Sal B for 24 h under normal culture conditions did not demonstrate toxicity (data not shown).

### Real-time polymerase chain reaction (PCR)

About 10^6^ cells were added to 1 mL Trizol (Invitrogen, Carlsbad, CA, USA) to extract total RNA. Total RNA (1 μg) was taken for reverse transcription using a SuperScript III Reverse Transcriptase kit with an oligo (dT) primer (Invitrogen). Expression of mature mouse miRNAs was determined by a stem-loop real-time PCR system using Maxima SYBR Green quantitative PCR Master Mix (Fermentas, Ontario, Canada) and StepOne Sequence Detector (Applied Biosystems, Foster City, CA, USA). The mmu-miR-30a-F2 primer was 5′-ACAGCCTGTAAACATCCTCG -3′ and the mmu-miR-30a-RT primer was 5′-GTCGTATCCAGTGCAGGG TCCGAGGTATTCGCACTGGATACGACTTCCAGT-3′. PCR primers for U6 were 5′-CTCGCTTCGGCAGCACATATACT-3′ and 5′-ACGCTTCACGAATTT GCGTGTC-3′. The universal primer downstream (mir-R2) was 5′-TCGTATCCAGT GCAGGGTC-3′. Data were normalized from control or I-R group (100 %) and expressed as a percentage of the control or I-R group.

### Western blotting

Expression of beclin-1, LC3-II, p-Akt and total-Akt in myocardial cells was detected by western blotting Protein samples were extracted from cardiac myocyte. Preparation of protein samples consisted of several steps, including splitting, centrifugation and boiling. Protein samples (20 μg) were fractionated by sodium salt (SDS)-Polyacrylamide gel electrophoresis (PAGE) (10 % or 12 % polyacrylamide gels), transferred to a polyvinylidene difluoride (PVDF) membrane and then blocked in 5 % bovine serum albumin and prepared in a Tris-buffered saline (TBS) for 1 h at room temperature. The membranes were incubated with specific antibodies (1:1000 dilution) against beclin-1, LC3-II, p-Akt, total-Akt and β-actin (Cell Signaling Technology, USA). Blots were detected using horseradish peroxidase-conjugated secondary antibody (1:2000 dilution; Santa Cruz Biotechnology, CA, USA) for 1 h at room temperature. After washing, the immunoreactive protein bands were developed by an Enhanced chemiluminescencekit (Pierce, Rockford, IL, USA) and the resulting membranes were imaged using the gel imaging system (BIO-RAD). The test of each protein was repeated 3 times. Data were normalized from control or I-R group (100 %) and expressed as a percentage of the control or I-R group.

### CCK-8 assay

Myocardial cells (1 × 10^3^ cells per well) were seeded on 96-well plates. The cells were either treated with I-R only, or treated with addition of Sal B. Normal cultured microglial cells without any treatment were used as control. Cell viability was evaluated with the CCK-8 assay, according the instruction of the manufacture . In brief,at the end of I–R, the medium in 96-well culture plates was changed to DMEM/F12 to avoid background interference. CCK-8 (10 μL) was added to each well followed by incubation for 2 h at 37 °C. A microplate reader was used to measure the optical-density value at 450 nm.. In control groups and experimental groups, 6 wells cells were observed and experiment was repeated 3 times. Data were normalized from control (100 %) and expressed as a percentage of the control group.

### LHD assay

For measurement of LDH leakage, he ischemia and reperfusion supernatants were collected. LDH activities were measured using the enzymatic reaction kinetics monitoring method according to the manufacturer’s instructions. The total LDH activity was obtained from adding LDH activities in the ischemia and reperfusion supernatants and the cell lysate together. Rate of LDH leakage was expressed using the following equation: Rate of LDH leakage = (OD value of the supernatant of the medium/OD value of the total cells) × 100 %. Data were normalized from control (100 %) and expressed as a percentage of the control group.

### Statistical analysis

Data were expressed as mean ± standard deviation (SD). Differences between experimental groups were examined by one-way analysis of variance (ANOVA), and means of two groups were compared using Student’s *t*-test (paired, 2-tailed) by SPSS 18.0 software. *P* < 0.05 was considered significant.

## Results

### MiR-30a expression decreases in myocardial cells with an I–R model and Sal B increases miR-30a expression

**Fig. 1:**
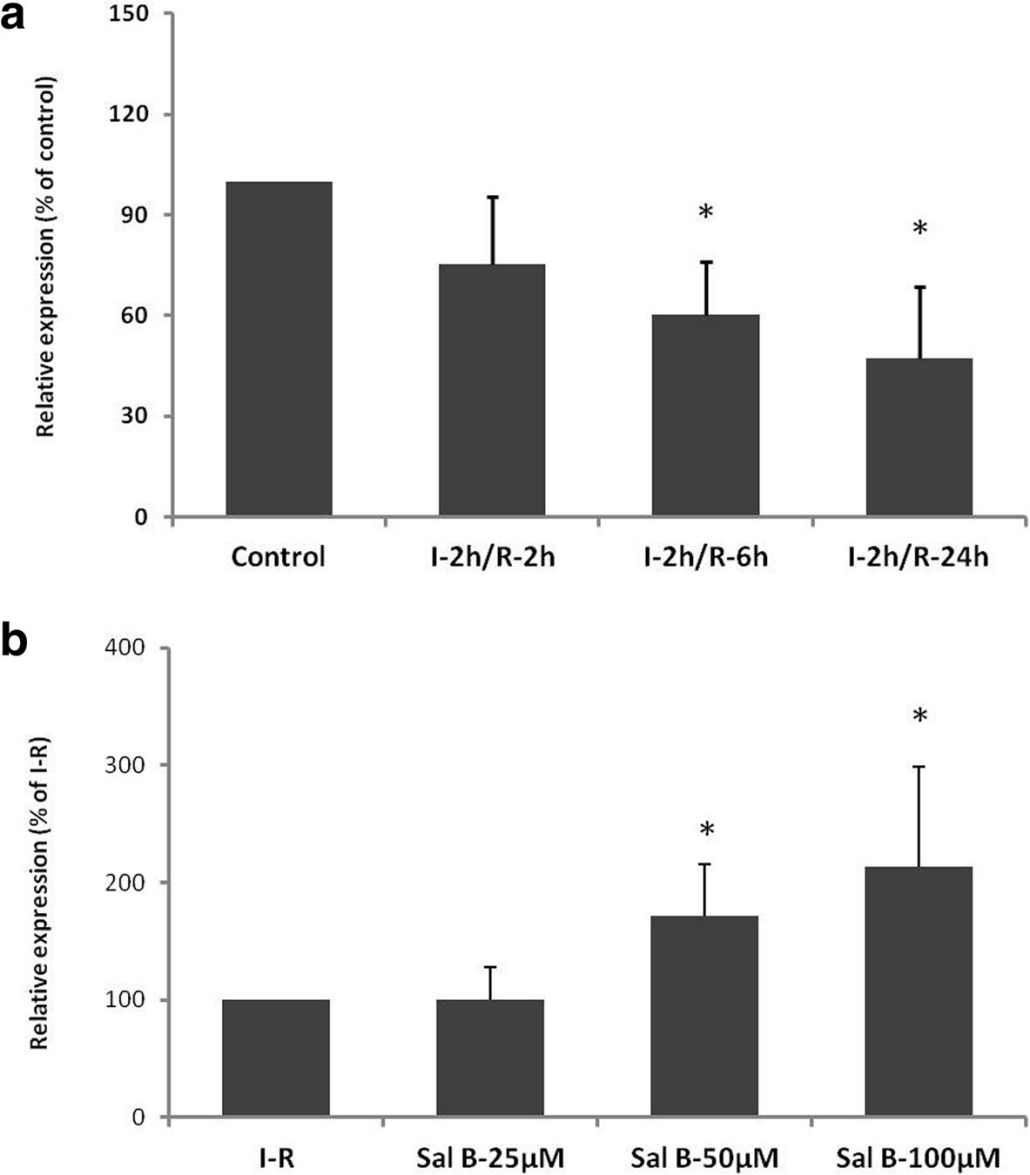
**a** MicroRNA-30a (miR-30a) level decreases in myocardial cells with I–R injury detected by real time-polymerase chain reaction (RT-PCR). I–R-2 h, I–R-6 h and I–R-24 h denote subjection of myocardial cells to reperfusion for 2, 6, and 24 h followed by ischemic injury, respectively. **b** Sal B increases miR-30a expression in a dose-dependent manner.. Values are the mean ± SD from 5 wells per group. *P <0.05 *vs* control or I–R-24 h group

Real-time PCR was first conducted to disclose the difference in miR-30a level between normal cultured cells and ischemic myocardial cells. Compared with the control group,the miR-30a level was reduced greatly in myocardial cells with I–R injury in a time-dependent manner, with the level in the I–R 24 h group (47.26 ± 21.33 %) being significantly different (*P* < 0.05; Fig. 1a). Compared with the I-R group, the miR-30a level could be improved by Sal B in a dose-dependent manner, with 50 μM (171.51 ± 43.49 %) and 100 μM groups (213.41 ± 85.68 %) showing remarkable changes (*P <* 0.05; Fig. 1b). According to the results shown above, we confirmed the I–R time to be 24 h and the dose of Sal B to be 50 μM.

### Sal B inhibits the autophagy of myocardial cells induced by I–R

**Fig. 2:**
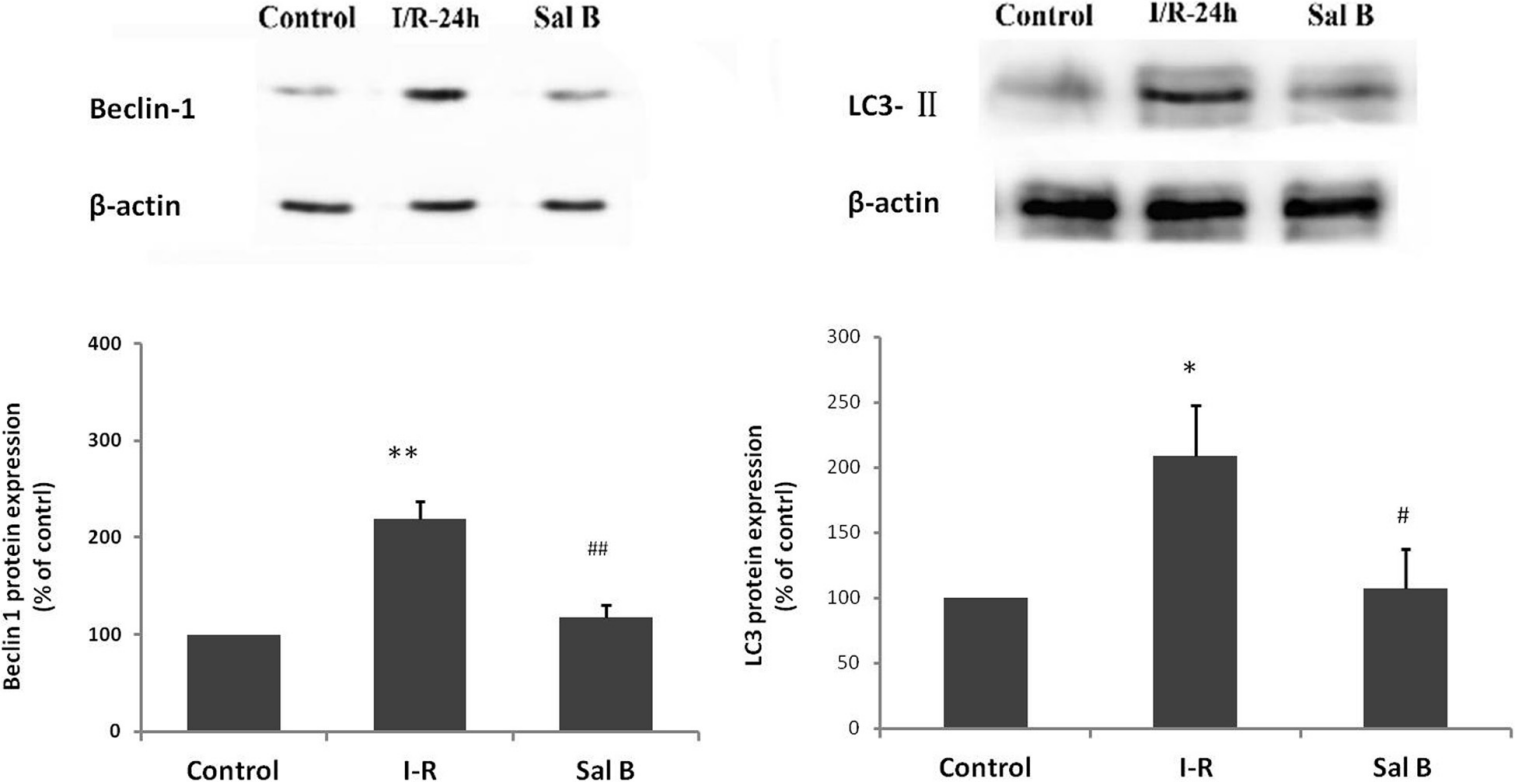
Sal B suppresses the levels of the autophagy-related proteins (**a**) beclin-1 and (**b**) LC3-II induced by I–R-24 h. Values are the mean ± SD from 3 wells per group. * *P* <0.05 *vs* control; **P <0.01 *vs* control; # *P* < 0.05 *vs* I–R-24 h group; ## *P* <0.01 *vs* I–R-24 h group

Based on the results of the time-course and dose-course experiments mentioned above, This study investigated the regulatory effects of 50 μM Sal B on autophagy in myocytes subjected to I–R for 24 h. Compared with the control group in this experiment, immunoblot analyses showed that I–R caused an increase in endogenous beclin-1 (218.81 ± 18.70 %, *P* < 0.01, Fig. 2a) and LC3-II (208.93 ± 39.19 %, *P <* 0.05, Fig. 2b), thereby suggesting increased autophagy. This increase was suppressed by Sal B, which suggested that increased autophagy in myocardial cells under I–R could be blocked by Sal B, with relative densities of beclin-1 and LC3-II of 117.47 ± 12.48 % and 107.52 ± 30.38 %, respectively.

### Sal B improves cell viability and reduces rate of LDH leakage of I–R-injured myocardial cells

**Fig. 3:**
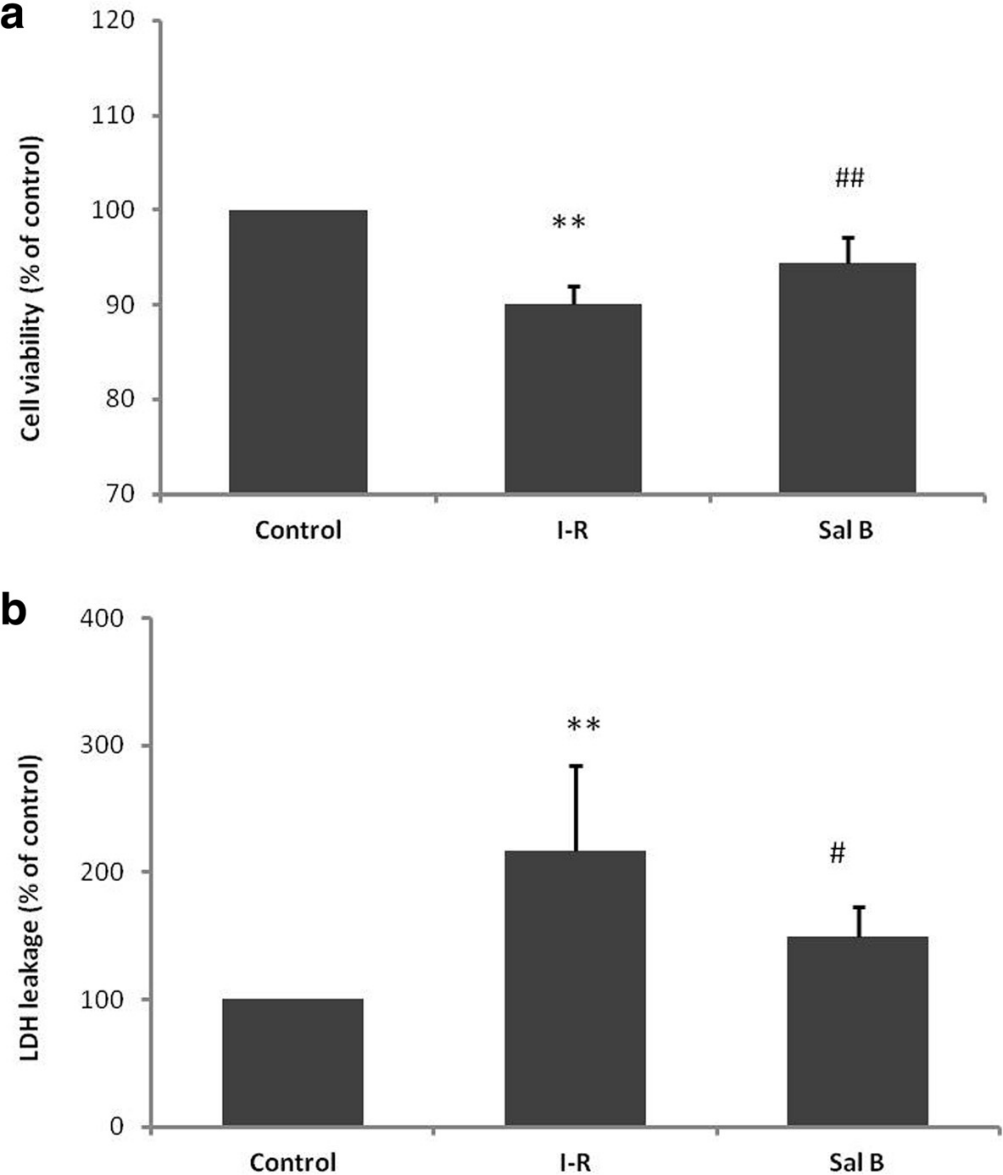
**a** Bar graphs show that Sal B increases the viability of cells subjected to I–R-24 h as determined by the CCK-8 assay. **b** Bar graphs show that Sal B reduces the rate of LDH leakage in the culture media of myocardial cells. Data are the mean ± SD from 6 wells per group. ** *P* <0.01 *vs* control; # *P* <0.05 *vs* I–R-24 h group; ## *P* <0.01 *vs* I–R-24 h group

Given that autophagy is non-selective and serves as a “double-edged sword” for ischemic injury in the heart, we next ascertained if the inhibitory effect of Sal B on myocardial autophagy was beneficial. A combination of cell viability and assay was used to measure the rate of LDH leakage, which are regarded as reliable markers of cellular injury. Figure 3a and b showed that compared with the control group,after cardiomyocytes were subjected to I–R for 24 h, significant suppression of cell viability (90.02 ± 1.87 %, *P* < 0.01) and LDH release were induced (217.22 ± 66.51 %, *P* < 0.01), which were recovered significantly by Sal B (94.41 ± 2.76 %, *P* < 0.01; 149.05 ± 24.35 %, *P* <0.05). These findings suggested that the inhibitory effect of Sal B on myocardial autophagy was beneficial for I–R-injured myocardial cells.

### Sal B-mediated anti-autophagy effect on cardiac cells is reversed by a miR-30a inhibitor

**Fig. 4:**
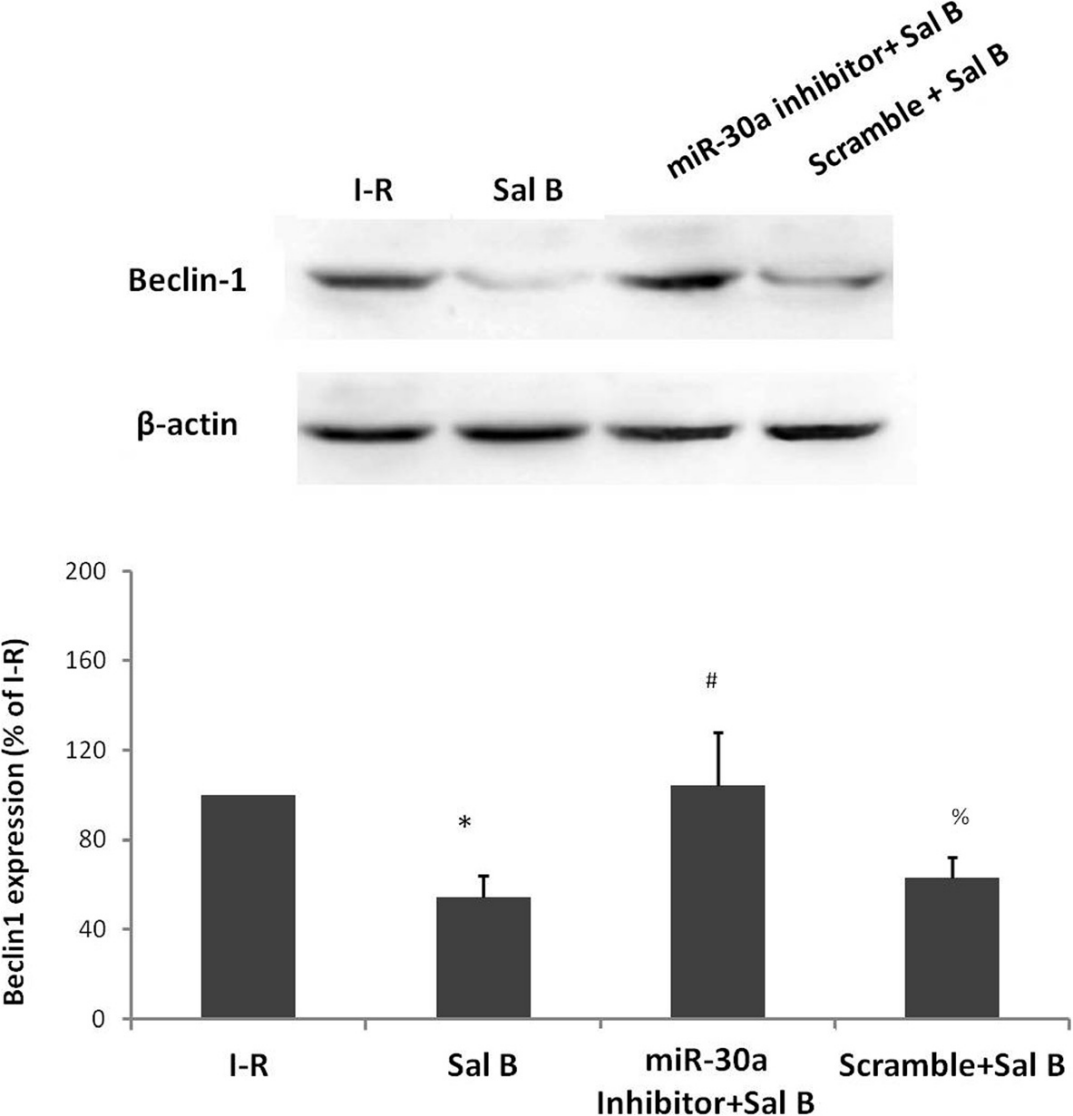
Sal B reduces the expression of beclin-1, which is reversed by a miR-30a inhibitor. Values are the mean ± SD from 3 wells per group. * *P* <0.05 *vs* I–R-24 h group; # *P* <0.05 *vs* Sal B group; % *P* <0.05 *vs* miR-30a inhibitor + Sal B group

Beclin-1 expression was evaluated to further confirm the mechanism of Sal B down-regulation of miR-30a-mediated cardiac autophagy (Fig. 4). Compared with the I-R group Sal B could significantly decrease beclin-1 expression (54.19 ± 9.96 %, *P* <0.05), suggesting suppression of autophagy. However, compared with the Sal B group, beclin-1 expression in the Sal B with a miR-30a inhibitor group increased by 49.8 %, whereas the scramble sequence had no significant effect on Sal B-induced cardiac protection against autophagy. These data suggested that Sal B-mediated miR-30a expression might protect myocardial cells against I–R injury, probably through regulation of autophagy.

### Inhibition of PI3K expression abrogates protection by Sal B-induced miR-30a expression

**Fig. 5:**
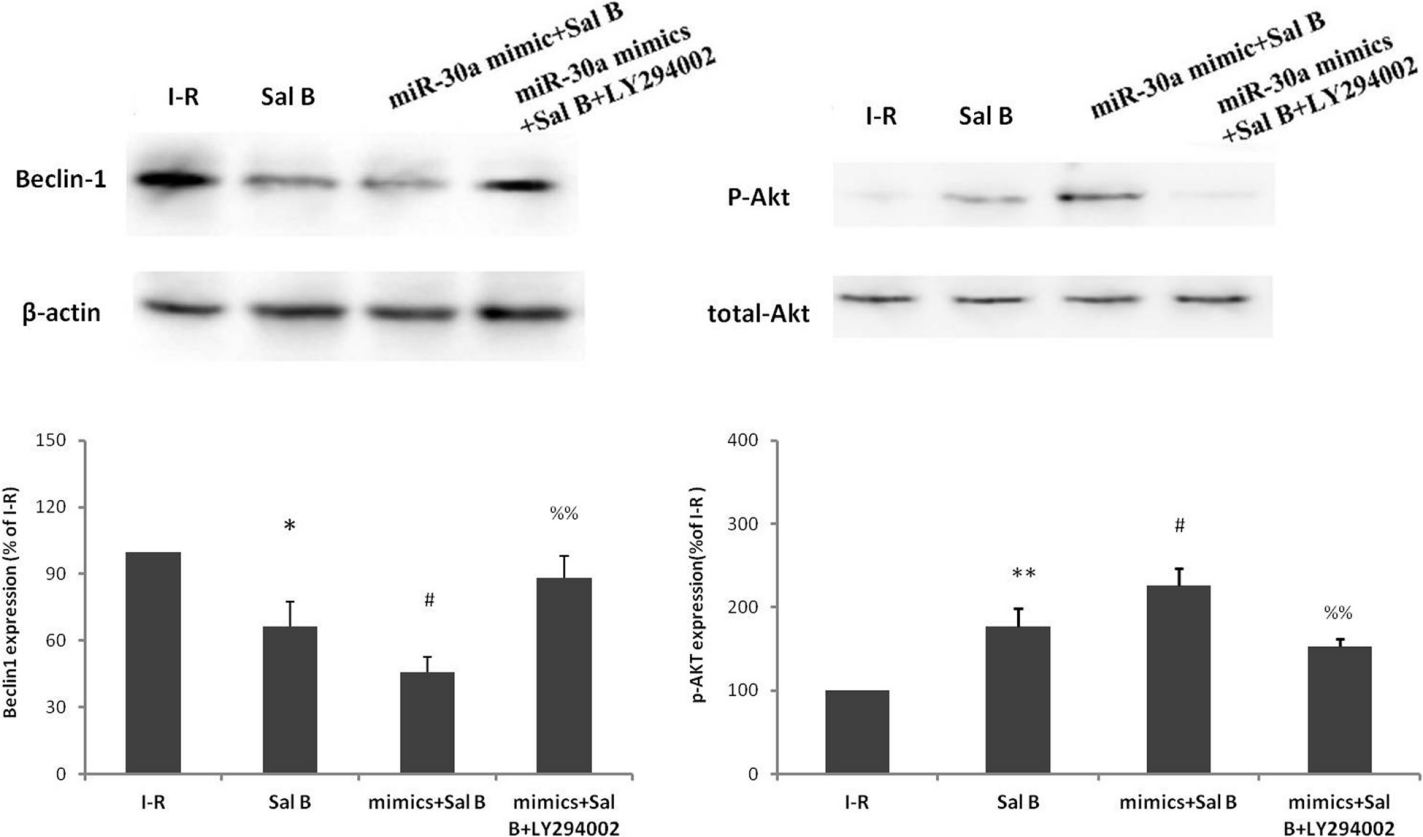
Modulations of expression of beclin-1 protein (**a**) and p-Akt (**b**) protein in mouse cardiomyocytes treated by miR-30a and the PI3K inhibitor LY294002 as determined by western blotting. Data are the mean ± SD from 3 wells per group. * *P* < 0.05 *vs* control; ** *P* <0.01 *vs* control; # *P* <0.05 *vs* Sal B group; %% *P* <0.01 *vs* miR-30a mimic + Sal B group

To demonstrate the direct link between the PI3K/Akt signaling pathway and miR-30a expression induced by Sal B, PI3K expression was inhibited using the PI3K inhibitor LY294002(5 μM), which was given for the first 15 min of I–R. Compared with the I-R group,treatment with Sal B could reduce beclin-1 expression (66.237 ± 11.22 %, *P* < 0.05; Fig. 5a) and induce p-Akt expression (176.954 ± 21.51 %, *P* < 0.01; Fig. 5b). No difference was observed in total Akt expression under different conditions (Fig. 5b). Overexpression of miR-30a could further increase expression of p-Akt to 226.253 ± 20.50 % and reduce beclin-1 expression to 45.505 ± 6.91 %. However, the PI3K inhibitor LY294002 could abrogate the cardioprotection of miR-30a against I–R.

## Discussion

Sal B is a natural compound used for the treatment of ischemic heart disease. Most studies of Sal B have focused on its ability to scavenge reactive oxygen species [26], inhibition of adherence between leukocytes and endothelial cells [27] and inflammation suppression [28]. Recent studies have revealed that miRNAs are involved in physiologic and pathologic processes in the cardiovascular system. The present study is the first to demonstrate that miR-30a expression can be mediated by Sal B, which plays an important in protection against cardiac I–R injury in vitro. The results presented here indicate that the potential signal pathway of Sal B inhibited miR-30a mediated cardioprotection might be achieved by targeting PI3K/Akt signaling pathway.

A subset of miRNAs are expressed abundantly in cardiac tissue, and have important roles in numerous cardiac diseases such as heart failure [29], myocardial fibrosis [30] and ischemic heart disease [31]. In diseases of the cardiovascular system, miR-30a plays an important part in regulation of autophagy through beclin-1 protein during myocardial injury induced by angiotensin II [32]. miR-30a expression has been reported to be increased in cultured myocardial cells [6] and in circulating plasma from patients with acute myocardial infarction [33]. In contrast, using quantitative real-time RT-PCR analyses, we found that miR-30a expression was down-regulated by I–R at 2, 6 and 24 h in a time-dependent manner. Specifically, the miR-30a level in myocardial cells subjected to I–R for 24 h appeared to decrease significantly. This finding suggested that reperfusion could suppress the miR-30a level (i.e., a high level of autophagy). This phenomenon could be suppressed by Sal B, which has not been reported before.

Several studies have focused on miR-30a target genes and their involvement in pathophysiologic processes. It has been demonstrated that miRNA-30a can bind directly with the 3′-UTR of *beclin-1* mRNA and promote degradation of its mRNA [34]. Beclin-1 is highly homologous with yeast autophagy-related gene 6 (Apg6/Vps30), which is essential for the induction and regulation of autophagy [35]. Studies have demonstrated that upregulation of beclin-1 expression promotes autophagic activity [36, 37]. Valentim et al. [38]. showed that inhibition of autophagy by genetic and pharmacologic inhibition of beclin-1 reduced death of cardiomyocytes subjected to simulated I–R. Given the increase in miR-30a expression by Sal B, we next observed that Sal B could suppress beclin-1 expression, suggesting inhibition of autophagy. LC3 is processed from LC3-I (16 kDa) to LC3-II (14 kDa), which is recruited to autophagosomes, and the increase in the LC3-II/I ratio is an indicator of up-regulated autophagy [39]. In addition to beclin-1, this study also observed that Sal B inhibited LC3-II expression, suggesting restriction of autophagic activity. The inhibition of autophagy induced by Sal B was induced by increases in miR-30a expression.

Whether autophagy has a protective or lethal role in the ischemic myocardium is controversial. In recent years, the regulation and contribution of the autophagic process to cell metabolism has been characterized in great detail. Several studies reported a role for autophagy in programmed cell death in heart disease. Paradoxically, some studies reported that in the case of myocardial ischaemic injury, autophagy causes cell survival, whereas the reperfusion injury causes cell death. It has been demonstrated that excessive autophagy leads to cell death. Thus, the extent of autophagy appears to be critical for determining whether it will play a protective or harmful role, This study wanted to know if suppression of autophagy by Sal B was beneficial for I–R-injured myocardial cells. Cell viability and LDH leakage from cells are used widely as reliable markers of cellular injury. The degree of LDH leakage is closely related to cardiomyocyte necrosis [40, 41]. Thus, the protective effects of Sal B on I–R-induced cardiomyocyte injury according to cell viability and LDH leakage was explored the protective effects of Sal B on I–R-induced cardiomyocyte injury according to cell viability and LDH leakage. Suppression of autophagy by Sal B was beneficial for survival of I–R-injured cells, as characterized by increased cell viability and reduced rate of LDH leakage. Next, we wished to ascertain if miR-30a was involved in the Sal B-mediated anti-autophagy effects on cardiomyocytes. After transferring a miR-30a inhibitor or the scramble sequence into cardiomyocytes, we found that the miR-30a inhibitor (but not the scramble sequence) could attenuate Sal B-induced cardioprotection against the autophagy induced by simulated I–R injury. Taken together, we deduced that Sal B protects cardiomyocytes through miR-30a upregulation-mediated inhibition of autophagy.

Consequently, this study will continue to identify the role of the PI3K/Akt signaling pathway in miR-30a-mediated autophagy after I–R injury in future studies. LDH release and cell viability was shown that I–R damaged myocardial cells and that, when I–R occurred, autophagy was increased as characterized by up-regulation of expression of beclin-1 and LC3-II. Sal B could reduce beclin-1 expression, and miR-30a mimics reduced beclin-1 expression further. After transferring a miR-30a inhibitor into the myocardium to “silence” expression of endogenous miR-30a, beclin-1 expression was increased, suggesting that miR-30a can protect myocardial cells through suppression of autophagy. When the PI3K inhibitor LY294002 was added into the system, beclin-1 expression was increased, suggesting that it abrogated the cardioprotective properties of miR-30a against I–R. From the viewpoint of autophagy,this study demonstrated (indirectly) that a PI3K inhibitor could abrogate the cardioprotective properties of miR-30a. Furthermore, inhibition of PI3K expression at the time of reperfusion abrogated p-Akt expression and the anti-autophagy effect of miR-30a induced by Sal B. Taken together, these data demonstrate that Sal B can alleviate I–R-injured myocardial cells through miR-30/PI3K/Akt pathway-mediated suppression of autophagy.

## Conclusion

In summary, our data suggest that miR-30a has a vital role in Sal B-induced cardioprotection. Upregulation of endogenous miR-30a expression induced by Sal B can alleviate I–R-induced myocardial autophagy, and the mechanism of action could involve regulation of the PI3K/Akt signaling pathway.

## Additional file

Additional file 1: Availability of supporting data. (XLSX 14 kb)

## Abbreviations

CCK-8: Cell counting kit 8
I/R: Ischemia/reperfusion
LDH: Lactic acid dehydrogenase
miRNA: MicroRNA
PI3K: Phosphoinositide 3-kinase
Sal B: Salvianolic acid B
